# The Intricacies of Computational Electrochemistry

**DOI:** 10.1021/acsenergylett.5c00748

**Published:** 2025-08-08

**Authors:** Nitish Govindarajan, Georg Kastlunger, Joseph A. Gauthier, Jun Cheng, Ivo Filot, Arthur Hagopian, Heine Anton Hansen, Jun Huang, Piotr M. Kowalski, Jinwen Liu, Juan M. Lombardi, Mikael Maraschin, Andrew Peterson, Hemanth S. Pillai, Hector Prats, Conor J. Price, René van Roij, Jan Rossmeisl, Ranga Rohit Seemakurthi, Seung-Jae Shin, Audrey Smith, Jia-Xin Zhu, Katharina Doblhoff-Dier

**Affiliations:** † School of Chemistry, Chemical Engineering and Biotechnology, 54761 Nanyang Technological University, 21 Nanyang Link, Singapore, 637371 Singapore; ‡ Catalysis Theory Center, Department of Physics, 5205 Technical University of Denmark (DTU), 2800 Kgs. Lyngby, Denmark; ¶ Department of Chemical Engineering, 6177 Texas Tech University, Lubbock, Texas 79409, United States; § State Key Laboratory of Physical Chemistry of Solid Surfaces, iChEM, College of Chemistry and Chemical Engineering, 12466 Xiamen University, Xiamen 361005, China; ∥ Laboratory of AI for Electrochemistry (AI4EC), IKKEM, Xiamen 361005, China; ⊥ Institute of Artificial Intelligence, 12466 Xiamen University, Xiamen 361005, China; # Laboratory of Inorganic Materials and Catalysis, Department of Chemistry and Chemical Engineering, Eindhoven University of Technology, 5600 MB Eindhoven, The Netherlands; @ Leiden Institute of Chemistry, Leiden University, PO Box 9502, 2300RA Leiden, The Netherlands; △ Department of Energy Conversion and Storage, Technical University of Denmark, 2800 Kgs. Lyngby, Denmark; ∇ Forschungszentrum Jülich GmbH, Institute of Energy Technologies, Theory and Computation of Energy Materials (IET-3), 52425 Jülich, Germany; †† 28259 Fritz-Haber-Institut der Max-Planck-Gesellschaft. Faradayweg 4-6, 14195 Berlin, Germany; ‡‡ School of Engineering, 6752 Brown University, Hope Street, Providence, Rhode Island 02912, United States; ¶¶ Department of Energy Conversion and Storage, Technical University of Denmark, DK-2800 Kgs. Lyngby, Denmark; §§ Department of Chemistry, Physical and Theoretical Chemistry Laboratory, University of Oxford, South Parks Road, Oxford OX1 3QZ, U.K.; ∥∥ Institute for Theoretical Physics, Utrecht University, Princetonplein 5, Utrecht 3584 CC, The Netherlands; ⊥⊥ Center for High Entropy Alloy Catalysis, Department of Chemistry, University of Copenhagen, Copenhagen DK-2100, Denmark; ## Institute of Chemical Research of Catalonia (ICIQ-CERCA), 202569 The Barcelona Institute of Science and Technology, Av. Països Catalans 16, Tarragona 43007 Spain; @@ School of Energy and Chemical Engineering, 131639 Ulsan National Institute of Science and Technology (UNIST), Ulsan 44919, Republic of Korea

## Abstract

Computational electrochemistry is hard—anybody who has ever tried will know. We argue that the reasons for its complexity lie not only in the multiscale nature of electrochemical processes but also in the rapid, ongoing method development in the field. This has resulted in a lack of clear guidelines and many open discussions in the community. These issues were also the topic of a recent Lorentz Center workshop, the key take-away messages of which are highlighted in this Perspective. In particular, we discuss why the choice between constant potential and constant charge simulations is less trivial than it may seem, why interpreting electrochemical reaction free energy diagrams can be challenging, why the Poisson–Nernst–Planck equation is not all there is, and why we desperately need more benchmarking in the field.

## Graphical abstract

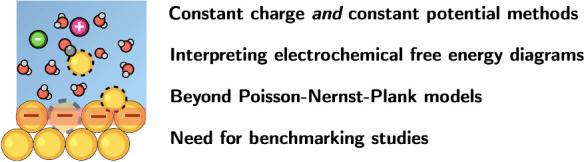

In the past few decades, there has been a rapid development of new computational methods in the field of electrochemistry. This development is driven by the important role of electrochemical processes in the transition toward a fossil-carbon free economy. Rapid method development is also inherently driven by the necessity of tackling the complex and dynamic nature of electrochemical processes.

To date, one cannot capture all aspects of the complexity of electrochemical systems in a single simulation. Consequently, one and the same electrochemical system is often addressed from various angles, with each approach offering distinct advantages and limitations. However, our current approaches generally rely on so many assumptions and approximations that our understanding of their true limitations can remain blurred. The lack of clarity in the best practices to study electrochemical processes, the inherent (multiscale) complexity of the problem, and the rapid development of methods make computational electrochemistry difficult and increases the likelihood that methods are used in situations where they are not appropriate.

To discuss the aforementioned issues, we organized a Lorentz center workshop on *Multiscale modeling of electrochemical processes* ^1^ where 46 primarily computational electrochemists with modeling expertise across different length and time scales came together to discuss pressing issues and new developments in the field. We would like to highlight key discussion points that emerged throughout the workshop. In particular, we will discuss why the choice between constant potential and constant charge ensembles in atomistic simulations is less trivial than it may seem (see highlight 1), why interpreting electrochemical reaction free energy diagrams can be challenging (see highlight 2), why the Poisson–Nernst–Planck equation is not all there is to model transport (see highlight 3), and why there is an urgent need for more benchmarking in the field (see highlight 4). For each of these points, we also provide a perspective on developments required to move the field forward.

## Electrochemical Processes Are Inherently Multiscale in Nature

In electrochemistry, experimental observations are often complex to interpret, as the physical origin of an observed effect can be the result of various physicochemical phenomena occurring across a wide range of length and time scales (Figure 1): mass transport, double layer effects, surface diffusion, adsorption/desorption, and electrode restructuring can each have their own influence on electrochemical processes.

**Figure 1:**
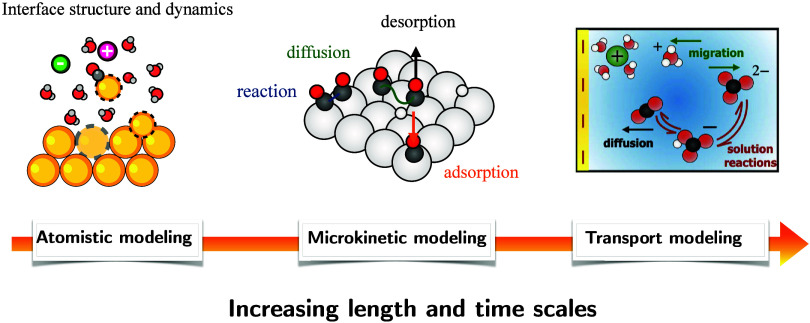
Schematic of the various simulation types (atomistic, microkinetic, and transport modeling) with increasing length and time scales needed to study electrochemical processes.

It is currently not feasible to capture all the relevant length- and time-scales in a single simulation. The general practice is therefore to focus on a single length/time scale and to leverage knowledge from one scale to the next, resulting in information flow both up and down the spatiotemporal scales. However, due to the convoluted nature of effects across the different scales, separating scales can lead to erroneous predictions and insights.

In the following, we will gradually ascend the space and time scales. This discussion will form a framework in which we highlight some of the key points that were raised during the workshop and some key take-away messages.

### Electrochemical Interfaces at the Atomic Scale

To obtain mechanistic insights into (electro)­chemical reactions occurring at the electrode surface, we generally have to resort to simulations at the atomic level. Two main challenges for atomistic simulations discussed during the workshop were (a) identifying the atomic structure of the electrode surface under *operando* conditions and (b) identifying appropriate approaches to treat charge transfer reactions in electronic structure simulations of the electrochemical interface and understanding their restrictions.

### *Operando* Electrode Structure

Electrochemical reactions strongly depend on the *operando* structure of the electrode (catalyst). Unfortunately, this (dynamic) structure is often hard to characterize using experimental techniques.

In modeling, thermodynamic stability descriptors are often used to identify the most stable state of the electrode^2–7^ or the structure of a nanoparticle under potential control.^8–10^ The *operando* structure of an electrode is, however, not uniquely defined by thermodynamic quantities (pressure, temperature, pH, bulk ion concentration, and potential). Instead, the entire (experimental) history plays a role. For example, surface reconstruction can be influenced by the potential range during cycling,^11^ requiring a kinetic description of the surface reconstruction.

In modeling, these kinetic factors are often not taken into account. This issue was discussed several times during the workshop and triggered interesting discussions as to whether the most stable state obtained using thermodynamic descriptors represents an appropriate starting point for further investigations. Additionally, it was mentioned that, when accounting only for the most stable structure(s), one might overlook the presence of minority surface sites, which can dominate the overall activity of a catalyst.^7^

In addition to the surface structure, leaching, corrosion, and the incorporation of solution-phase species can also affect the interfacial composition and the reactivity of an electrocatalyst. A good example is SrIrO_3_, which is one of the best reported catalysts for oxygen evolution—but only after selective leaching of Sr, leading to the formation of a highly active IrO_*x*_/SrIrO_3_ phase.^12^ To investigate the thermodynamic stability of a catalyst, traditional Pourbaix diagrams can be used. In the context of electrochemistry, the thermodynamic driving force for leaching and corrosion of charged species is generally computed via a Born–Haber cycle, relying on experimental equilibrium potentials.^13,14^

Unfortunately, traditional Pourbaix diagrams do not account for the presence of an interface, which can affect the stability of a catalyst. A well-known example, in which the sole consideration of bulk stability is insufficient, is aluminum. Aluminum would corrode (and ultimately dissolve) unless passivated by a stabilizing surface oxide layer. To bridge the gap between understanding the stability of the bulk and the surface of an electrode, the concept of a “surface Pourbaix diagram” was developed. Surface Pourbaix diagrams consider a constrained equilibrium surface coverage of intermediates at a given potential and pH^15^ but generally do not consider leaching and corrosion, limiting their predictive power. Additionally, and as mentioned above, reconstruction, adsorption, leaching, and corrosion are ultimately driven by kinetic considerations that are not captured in thermodynamic descriptors.

Despite advances in understanding the stability of catalytic materials in electrochemical environments, identifying and modeling the true active sites and reconstruction pathways of dynamic electrocatalyst surfaces under reaction conditions remain critical challenges. We anticipate that machine learning procedures will play an important role in improving this situation.^16,17^

### Reaction Thermodynamics

The main force driving an electrochemical reaction is the applied potential. Atomistic models of a catalytic surface alone are not sufficient to account for the potential. Therefore, any computational method would *a priori* require the description of the entire double layer. This requirement is circumvented by the computational hydrogen electrode (CHE) method that uses an elegant thermodynamic framework to account for the applied potential.^18^ Unfortunately, the CHE method cannot deal with situations involving partial charge transfer as commonly encountered in multistep electrochemical reactions. In addition, the electrolyte can also have a direct influence on electrochemical reactions.^19–23^ To date, electrolyte effects and the explicit buildup of a potential are mostly addressed via mean-field approaches. In this case, local effects and specific interactions are lost. Alternatively, one can explicitly include a fraction of the electrolyte. To fully account for the liquid nature of the electrolyte, *ab initio* molecular dynamics and free energy sampling are required, leading to a high computational cost, often necessitating harsh approximations. Computationally less expensive, quantum mechanics/molecular mechanics (QM/MM) based approaches have also been used^24^ but come with their own challenges.[Fn fn1]

Overall, the correct incorporation of electrolyte effects, including specific interactions, local electric field effects, and sufficient sampling under the correct thermodynamic conditions (see below), remain a challenge though—a fact that triggered critical questions throughout the workshop. We will discuss this issue in more detail in the next section.

### Electrochemical Barriers

In the community, there is no consensus on the correct (or optimal) approach to estimate electrochemical reaction barriers. Although we cannot resolve the issue here, we want to summarize some of the key points driving this ongoing discussion.

Similar to reaction thermodynamics, electrochemical barriers depend on the applied potential (or, equivalently, the corresponding interfacial electric field). Changes to barrier energies *G*°^‡^(*U*_abs_) with potential are often quantified in terms of a symmetry factor, β in a linear-like manner relative to the barrier obtained at an absolute reference potential *U*_abs_^1^ as

1 $$G^{{}^{\circ}‡}\left(U_{abs}\right)=G^{{}^{\circ}‡}\left(U_{abs}^{1}\right)+\beta \left(U_{abs}\right)\cdot \left(U_{abs}-U_{abs}^{1}\right)$$

Nonlinearities are captured by allowing β to be potential dependent. The symmetry factor β­(*U*_abs_) is, however, *a priori* not known, and can vary (typically between 0 and 1). This can lead to large errors in the estimate of *G*°^‡^(*U*_abs_)—in particular when (*U*_abs_ – *U*_abs_^1^) is large. Therefore, separate barrier calculations should be performed in the range of potentials of interest to estimate β.^25,26^

To compute potential-dependent electrochemical reaction barriers, various approaches are available as summarized in Figure 2. Constant potential methods^27–31^ are often preferred over constant charge or constant electric field methods.^32^ The reason may be that constant potential methods do not require extrapolation to the infinite cell size limit in order to account for the applied (and thus constant) potential.^33–35^ However, constant potential approaches also have their own limitations.

**Figure 2:**
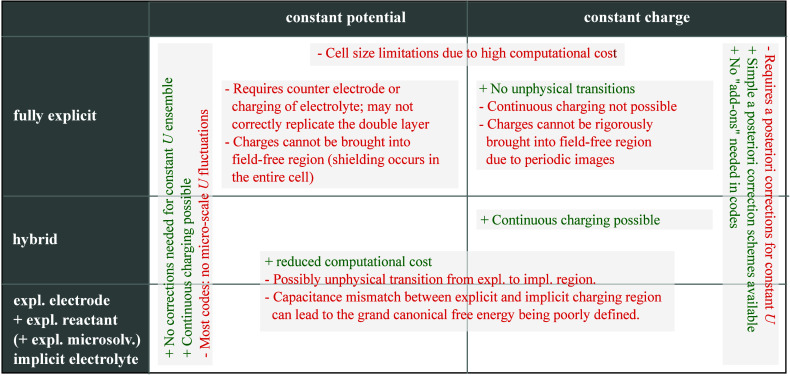
An overview of the advantages and limitations of the various constant potential and constant charge approaches available to compute electrochemical barriers. “fully explicit” and “implicit” refer to an atomistic and mean-field description of the electrolyte, respectively. See text for details.

First, one should ask whether individual electrochemical reaction events truly occur at constant potential. Santos and Schmickler^36^ have recently challenged this assertion based on the fact that time scales for double layer relaxation and the response time of the potentiostat can be much larger than the reaction time scale. Consequently, they argue that the reaction should proceed under constant charge conditions. It is debatable whether a single reaction event should actually change the potential—a macroscopic observable—or whether such a reaction-induced change in potential is an artifact of the periodic, relatively small unit cells used in DFT simulations. However, one fact remains: if the relaxation of solvent and ions are slow compared to the reaction time scale, reaction kinetics will be nonergodic,^37,38^ suggesting that, in these cases, the electrolyte should not be treated in thermodynamic equilibrium. As discussed in more detail in refs 37 and 39, this should not typically be an issue for activated processes with barriers above 0.2–0.3 eV in aqueous solvents but may play a role for non- or weakly activated reactions (as can be achieved by tuning the potential), large solutes, or in ionic liquids. Under these circumstances, conditions of thermodynamic equilibrium, as typically assumed in constant potential calculations (e.g., in JDFTx^30^ or the reference interaction site method^40^), are not necessarily met.

Second, and somewhat related to the issue above, at the microscopic scale, the potential (between a microscopic subpart of the surface and any position in space) as well as the surface charge fluctuate.^41^ This has to do with the *RC*-time scales of the capacitor and is similar to the temperature in an *NVT* ensemble, which should also fluctuate in small simulation cells. Potential fluctuations are not accounted for in most constant potential simulations. Adding them via a thermopotentiostat^42^ is possible but has restrictions as the time-scales of the fluctuations present in the real system are generally unknown. Additionally, in real systems, several physical effects causing fluctuations will play a role (e.g., surface conductivity, solvent fluctuations, ion diffusion, response of the potentiostat), causing fluctuations at different time-scales. To date, it is still an open question whether these fluctuations will affect relevant electrochemical reactions in an important way, but one may expect some influence, in particular, when reactions proceed fast enough to be able to react to the fluctuations.

Third, while constant potential methods guarantee the overall potential drop to be constant, the corresponding potential profile may be incorrect. In particular, most (fully) implicit solvation schemes grossly underestimate the double layer capacitance,[Fn fn2] neglect its potential dependence,^45^ and inaccurately predict the impact of electrolyte on the work function, as demonstrated in ref 44. This may contribute to an incorrect description of the interfacial electric field, which in turn can be expected to lead to inaccurate reaction energetics for adsorbed species with sizable dipole moments and for electrochemical barriers.

To obtain an improved description of the potential profile at the interface and to improve the description of the hydration of (species at) the interface, hybrid explicit + implicit (e+i) solvation schemes have been proposed.^44^ In e+i models, the implicit solvation and countercharge are used to keep the potential constant. The explicit electrolyte molecules, on the other hand, hydrate the interface. This can be done statically, by combining microsolvation with implicit solvation,^46,47^ or dynamically, by allowing the explicit solvent to move via molecular dynamics simulations.^48^ However, even in e+i schemes, certain caveats remain: (i) Depending on the solvation model, there may be an artificial potential drop at the explicit/implicit interface, either caused by artificial solvent alignment or a “gap” region. Such a potential drop will distort the relation between applied potential and surface electric field. The fact that e+i schemes often have a capacitance that is much lower (∼10 μF/cm^2^)^49–52^ than that found in fully explicit models^53,54^ is an important indication that such an effect could play a role. (ii) In e+i schemes, double layer screening occurs only in the implicit solvent region. In particular for a wide explicit solvent region, this can be an important restriction. As a consequence, an electric field is also present in the entire explicit region, and any charged species in this region cannot move into the field-free region, possibly requiring the use of pseudo-initial or pseudo-final states when describing electrochemical reactions (see next section). (iii) Depending on the solvation model, implicit electrolyte may unphysically “leak” in between explicit electrolyte molecules. Some commonly used polarizable continuum models have recently introduced “solvent aware” cavity formation definitions which largely mitigate this issue.^55^ (iv) Capacitance mismatch between the explicit and implicit electrolyte can lead to the grand canonical free energy being a poorly defined quantity at a given potential, manifesting as a finite cell-size effect.^56^

The above-mentioned issues explain why fully explicit interface models, which are intrinsically at constant charge, are sometimes preferred. Unfortunately, these methods come with their own challenges, as summarized in Figure 2: (i) The use of a constant charge ensemble in small supercells requires approximate correction schemes that rely on the extraction of the (quantum-mechanically ill-defined) surface charge^57^ or on the assumption that the charge rearrangement in the cell due to the reaction is equivalent to the charging of the double layer.^33,51^ (ii) The computational cost for such simulations is excessively large, resulting in the use of harsh approximations in the computational setups as discussed later in this article. And (iii), even in these approaches, a charged particle cannot be rigorously brought into a field-free region due to the periodic repetition of a (typically small) unit cell, leading to an effective capacitive charging between the charged species and the surface, which in turn results in an incorrect electrostatic energy response.[Fn fn3]

To summarize, while constant charge approaches and constant potential approaches are equivalent in the infinite size limit,^33–35^ each approach comes with its own approximations when used in practice. To date, it often remains unclear which of the approximations/omissions of a given model has the largest impact on the outcome of our simulations. It is our firm belief that, to ultimately find a consensus within the community as to the strengths and limitations of various methods, fundamental studies and (more)^57^ rigorous method comparisons are needed.

**Highlight 1:** Overall, we believe that it is not so much a matter of constant potential vs constant charge methods, but rather the matter of constant potential *and* constant charge methods, as both approaches have their strengths and limitations (see Figure 2).

We believe that future work should provide a better understanding of (i) how the (local or mean-field) surface electric field or potential affect the energetics, (ii) when the (approximate) correction schemes for constant charge simulations in finite system sizes^57^ break down, and (iii) which artifacts are caused by the use of small unit cells (e.g., overstructuring of water, unrealistic ion density profiles). The computational challenge or “hackathon” that was held during the Lorentz workshop was aimed at highlighting the need for such fundamental investigations, and raising awareness on issues related to the various approaches.

### Interpreting Electrochemical Free Energy Diagrams

Once the reaction barriers are known, reaction rates can be predicted using microkinetic modeling. A qualitative analysis can also be obtained directly by analyzing free energy diagrams. However, we observed during the workshop that this is not as simple as it might seem. Part of the difficulty stems from the fact that free energy diagrams of electrochemical reactions are presented in different ways, depending on the approach used and the preferences of the authors involved in the study.

Another issue is that the variables included in the Gibbs free energy are often not clearly defined, and the differentiation between Gibbs free energies and standard Gibbs free energies is not clearly made. Results obtained from a CHE-based approach are generally reported as the Gibbs free energy (Δ*G*) of the different elementary reaction steps at 0 V vs RHE (i.e., versus the reversible hydrogen electrode) or at the *U*_RHE_ value corresponding to equilibrium conditions (i.e., when the overall reaction free energy Δ*G*_rxn_ = 0). For barriers, however, one often plots the *standard* Gibbs free energy profile (Δ*G*°(*x*)) profile, i.e., the Gibbs free energy at standard conditions of all reactants and products as a function of the reaction coordinate *x*. This is a logical choice as this quantity is what “drops out” of a nudged-elastic band calculation[Fn fn4] and free energy sampling. However, the choice of *G* vs *G*° is often not clearly indicated. In addition, one should also distinguish between electronically grand canonical (*G*(μ_el_)) and electronically canonical (*G*(*N*_el_)) energies. Various constant potential methods provide electronically grand-canonical energies as output, and the CHE and constant charge methods (with and without using correction schemes, such as the one proposed by Chan and Nørskov^57^)­[Fn fn5] are also consistent with electronically grand canonical energies, but the distinction between *G*(μ_el_) and *G*(*N*_el_) is not always clearly made in papers. Finally, for electrochemical reactions or reactions containing species with a strong dipole, the (standard) Gibbs free energy profile will depend crucially on the applied potential versus vacuum or (equivalently) the initial surface charge. It should therefore go without saying that these conditions should be clearly indicated, which is, unfortunately, not always the case, and often such diagrams are (incorrectly) assigned to *U* = 0 V vs RHE or SHE (by the reader).

**Highlight 2:** Electrochemical literature would benefit from a clearer nomenclature: Clear distinctions between Gibbs free energies *G*, standard Gibbs free energies *G*°, and electronically grand canonical energies *G*(μ_el_) vs electronically canonical energies *G*(*N*_el_) are needed. Additionally, the potential dependence of electrochemical free energy diagrams should be recognized and the potential (or surface charge), which they are estimated at, should be clearly stated.

An additional challenge in correctly interpreting Gibbs free energies obtained directly from barrier calculations is that charged reactants or products in solution (e.g., H^+^ or OH^–^) are generally not accurately captured in current computational approaches, as they often cannot be brought into a “bulk solution” region. Instead, calculations often suffer from (i) charge transfer to and from species that reside (too) close to the interface (offsetting the chemical energy at this “initial” (or “ final” state) and (ii) the fact that the charged species do not reside in a field-free region (offsetting the electrostatic energy of the species) (see Figure 3). We refer to such states with residual interactions to the catalyst surface as “pseudo-initial” or “pseudo-final” states. Considering such a pseudo-state as the initial or final state is equivalent to restricting the range of the reaction coordinate, such that the full elementary step will not be captured (see Figure 3b). This can lead to incorrect reaction barrier predictions (see Figure 3b) and reaction free energies that generally do not scale correctly with the applied potential, *U*. Unfortunately, when presenting free energy diagrams, this possible issue is often not considered: In typical AIMD simulation setups it is, strictly speaking, not even possible to bring a charged species into a field-free region (see also Figure 2). Similarly, in hybrid explicit + implicit methods, bringing charges into a field-free region is difficult because it would require moving the charged species from the explicit region to the implicit region. This would likely introduce other errors due to an unbalanced description of the solvation in the explicit and implicit regions. In how far the resulting pseudo-initial/final states will affect the actual result will depend on the exact computational setup. We assert that good practice requires one to check for the possible presence of pseudo-initial or pseudo-final states. This can be achieved by studying a reaction that is expected to be well described by the CHE. By comparing reaction free energies and their dependence on the potential and pH as obtained using the CHE framework and the computational scheme in question, one should be able to identify issues arising from the presence of pseudo-states.

**Figure 3:**
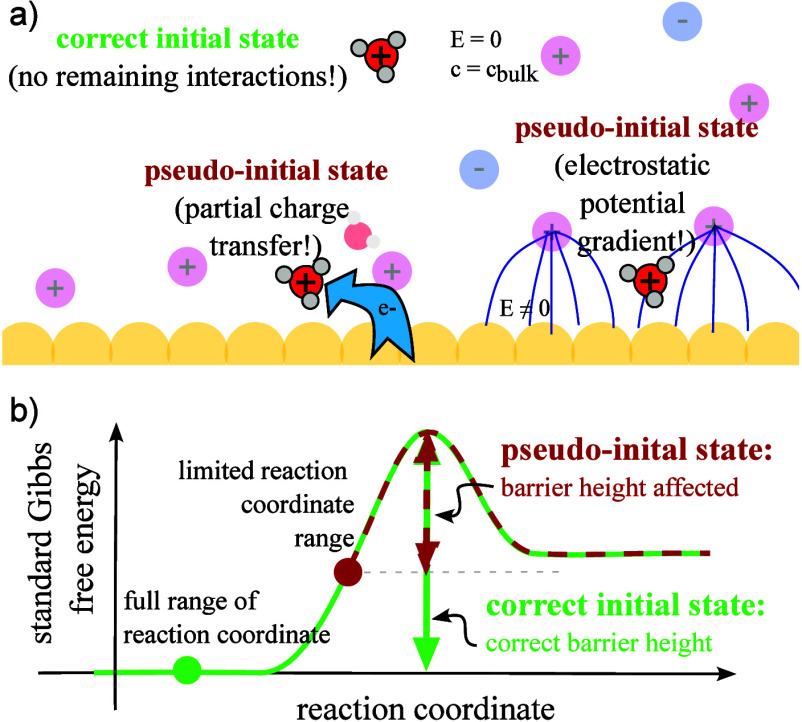
a) Schematic of possible causes for pseudo-initial states in simulations. The true initial state is characterized by the ion residing in a region where it does not interact with the electrode and the electric field is zero. For particles residing (too) close to the interface, charge transfer and electrostatic energy gradients can affect the reactant state and its energy. b) Sketch of a free energy diagram when using a correct initial state (green) vs a pseudo-initial state (red). The use of a pseudo-initial state will lead to incorrect barrier predictions and incorrect reaction free energies.

If a simulation is affected by the presence of a pseudo-state, one can choose to correct for it *a posteriori* by adjusting the initial or final states to those obtained from the CHE-model or, generally speaking, from a Born–Haber cycle. As an example, the Gibbs free energy of the pseudo-initial state *G*(H^+^@int|_*U*_abs__) in an acidic Volmer step should be replaced by

$$G\left({H^{+}}_{\left(bulksol\right)}+\mathrm{int}{|}_{U_{abs}}\right)=G^{{}^{\circ}}\left(\mathrm{int}{|}_{U_{abs}}\right)+\frac{1}{2}G^{{}^{\circ}}\left(H_{2}\right)-eU_{RHE}$$

where $U_{RHE}=U_{abs}-U_{SHE,abs}+\frac{k_{B}T}{e}\;\ln\left(10\right)pH$ and *U*_SHE,abs_ ≈ 4.44 V is the absolute potential of the standard hydrogen electrode.[Fn fn6] This has been done, for example, in refs 25 and 59–62 .[Fn fn7] Obviously, this method will lead to errors whenever a reaction is ill-described within the CHE method. However, depending on whether the charged state is the reactant or product, such a shortcoming might not necessarily impact the magnitude of the barrier.

### Interpreting Multistep Reactions

While reaction barriers are typically extracted in plots showing the standard free energy (see above), reaction networks and multistep reactions are often easier to analyze when plotting Δ*G* ^‡^ rather than Δ*G*°^‡^. Using Δ*G* ^‡^, the reaction rate of elementary steps involving bulk species can be written without explicit reference to the bulk concentrations

2 $$r=A\cdot \underset{i}{\prod }{\left[i\right]}^{\nu_{i}}\cdot e^{-\Delta G^{{}^{\circ}‡}/k_{B}T}=A\cdot e^{-\Delta G^{‡}/k_{B}T}$$

which facilitates the identification of an “effective” barrier Δ*G*_eff_^‡^ that can be used to approximate the overall reaction rate (*r*) of a multistep reaction as

3 $$r\propto e^{-\Delta G_{eff}^{‡}/k_{B}T}$$

It follows from textbook kinetics that Δ*G*_eff_^‡^ can be approximated by the largest free energy difference between any transition state (TS) and the Gibbs free energy of the resting state prior to this TS. This approximation will be reasonable as long as the adsorbate coverage in the resting state is stable. By comparing Δ*G*_eff_^‡^ for different reaction pathways, the most probable reaction pathways can be identified directly from the free energy diagrams. Figure 4 illustrates how to determine Δ*G*_eff_^‡^ using the example of an electrocatalytic oxidation reaction. As can be seen, it is not (necessarily) the elementary step with the highest activation energy that defines the rate of a reaction. Instead, it may be a series of steps that determine the effective barrier. The traditional limiting potential analysis,^18,63^ which is still widely used, does not account for this possible combined effect of several reaction steps. As a consequence, the limiting potential analysis is also unable to explain certain trends in product selectivity, for example in the oxygen reduction reaction^48,64^ and CO_2_ reduction.^65^ Although extremely simple and powerful, traditional limiting potential analysis should therefore be used with caution.[Fn fn7]

**Figure 4:**
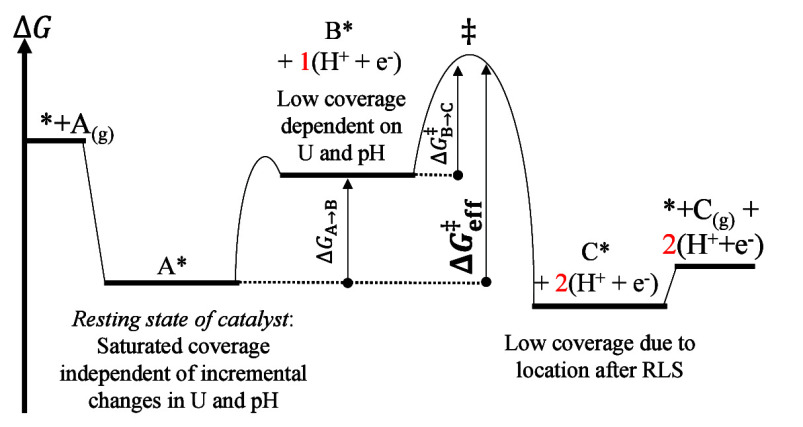
A sample free energy profile for a two-step electrocatalytic oxidation of A_(g)_ to C_(g)_ with the effective barrier Δ*G*_eff_^‡^ approximated as the largest free energy difference between the transition state and the resting state (A*). The exergonic adsorption of A_(*g*)_ results in a sizable coverage of A*, making it the resting state of the catalyst. The free energy of B* is irrelevant to the rate of the reaction as long as Δ*G*_A→B_ > 0 and its barrier is not as high as that of the next step. Finally, the free energy of C* also does not affect *r*, as it is located after rate-limiting step of the reaction.

A characteristic of electrochemical free energy diagrams is not only that they can be used to predict effective barriers and the most probable reaction pathways but also that the same diagrams can also be used to predict Tafel slopes and pH dependencies of the reaction rate.^52,66^

Within the effective-barrier approximations, the Tafel slope is given by

4 $${{\left(\frac{\partial \log\left(r/r_{0}\right)}{\partial U}\right)}^{-1}|}_{pH}=\frac{\ln\left(10\right)k_{B}T}{N_{ec}+\beta_{RLS}}$$

where *N*_ec_ is the number of equilibrated steps involving the creation or annihilation of an ion–electron pair (including proton–electron pairs) between the resting state and the rate-limiting step, and β_RLS_ is the symmetry factor.^52,66^ [Fn fn8]

The pH dependence is given by

5 $${\frac{\partial \log\left(r\right)}{\partial pH}|}_{U_{abs}}=\pm N_{pe}+N_{{OH}^{-},ini}-N_{H^{+},ini}$$

where ± refers to oxidation (+) or reduction (−), *N*_pe_ is the number of equilibrated steps involving the creation or annihilation of proton–electron pair *only* between the resting state and the rate-limiting step, and *N*_OH^–^_ _,ini_(N_H^+^__,ini_) are the number of OH^–^(H^+^) ions involved as reactants in the rate limiting step.^52^

These relations can, on the one hand, be used to verify theoretically predicted reaction mechanisms by comparing them to experimental results where available but can, on the other hand, also serve to extract mechanistic insight (e.g., the number of involved ion–electron transfer pairs vs the number of involved proton–electron transfer pairs and the symmetry factor) directly from experimental results. In this regard we note, though, that experimental Tafel slopes should be treated with care as they are relatively prone to errors due to mass transport limitations and bubble formation.^69^

Overall, it is clear that even such a qualitative analysis of free energy diagrams can provide important insights into electrochemical reaction kinetics. To allow for a reuse and reanalysis of data provided in papers, we repeat, however, our earlier statement that care should be taken to clearly indicate what is plotted [e.g., *G*° vs *G* and *G*(μ_el_) vs *G*(*N*_el_)]. Additionally, it is important to ensure consistency throughout the free energy profile (i.e., keeping the number of particles involved constant throughout the entire free energy diagram).

### Microkinetic Modeling

Whenever a qualitative assessment outlined in the previous section is not sufficient to predict reaction rates or understand reaction mechanisms,[Fn fn9] one needs to resort to microkinetic (MK) modeling.

In spite of the difficulty in obtaining reliable reaction barriers from first-principles based approaches, both “bottom-up” models^52,60,70–72^ (using barriers obtained from first-principles calculations) and “top-down” models (in which certain model parameters are fitted to reproduce experimental results) are used to understand reaction kinetics.^70,73^ The latter approach goes back more than half a century^74^ and has been used successfully to rationalize various experimental observations in electrocatalysis.^75–78^ A difficulty, however, is to make a decision on the complexity of the model employed. A fundamental choice lies in the description of the reaction network (e.g., as one, overall reaction with an effective barrier, or using individual reaction steps), and the description of the potential dependency (e.g., via Butler–Volmer type relations, Marcus theory, or by including potential-dependent barriers from DFT calculations). Another choice in the complexity of the model lies in whether parallel reaction networks are considered to account for multiple active sites or (mean-field) lateral interactions between adsorbates. Deciding on the choice of the MK model without detailed insights from first-principles calculations is nontrivial, and it seems to be “best practice” to keep the model as simple as possible so that they are computationally tractable and to avoid potential overfitting.^79,80^

Mean-field MK models typically struggle in systems where spatial correlations play an important role. Examples include island formation, steric exclusion effects (including those caused by multidentate species), complex reaction patterns involving adsorbates in specific arrangements or oscillatory dynamics, such as the formation of complex, long-range spatiotemporal patterns.^81^ Accounting for such effects requires moving beyond mean-field approaches toward kinetic Monte Carlo based methods.^82,83^

### Mass Transport and Electrokinetic Phenomena

Measured current densities during electrochemical processes often depend on the mass transport properties of the reactor. The simplest model to account for mass transport effects is a (1D) reaction–diffusion model that accounts for Fickian diffusion of species across a boundary layer. To further account for electromigration of charged species (e.g., ions) that are relevant in the formation of the electrical double layer, Poisson–Nernst–Planck (PNP) and its size-modified version (GMPNP) have been used in a number of studies.^84,85^ These models can further be coupled to reaction fluxes obtained from ab initio based microkinetic models and include solution phase reactions (e.g., buffer reactions in CO_2_R) in a self-consistent manner to develop “bottom-up” multiscale models.^85,86^ Although diffusion and electromigration have been accounted for in previous studies, discussions during the workshop highlighted that electrokinetic phenomena are not generally considered.

**Highlight 3:** In computational electrochemistry, electrokinetic transport phenomena are often neglected despite their potential relevance.

Electrokinetic phenomena include electro-osmotic flow (fluid flow due to a body force on the nonzero charge density of mobile ions in the electric double layer) and streaming currents (electric currents caused by a pressure-induced flow of mobile charges in the electric double layer). These effects could play a significant role in transport-dominated regimes of electrochemical processes. Recent studies show that fluid flow can directly couple to the chemical processes at the surface,^87^ resulting in initially homogeneous surfaces to become heterogeneous with a charge-density gradient in the flow direction.^88,89^ Similarly, an applied potential drop across cone-shaped channels (which are omnipresent in many porous materials) can yield ionic accumulation and depletion in the channel depending on the polarity of the applied voltage,^90,91^ and can thereby affect the surface chemistry.^92^ Exploring these electrokinetic and iontronic effects might thus be important in the development of multiscale models.

To ultimately compare the results from modeling studies to experiments and develop accurate insights into electrochemical processes, it is essential to transition from modeling reaction kinetics to *reactor kinetics* that requires the consideration of complex mass transport and electrokinetic phenomena in multiscale models of electrochemical processes.

## Benchmarking Computational Approaches

To identify the similarities, differences, strengths, and weaknesses of the various (multiscale) modeling approaches currently available, thorough method comparisons are urgently needed. During the workshop, the possibility of curating a standardized test set was discussed. However, we identified several issues with the idea of a “universal benchmark”.

First, for most electrochemical processes, the “ground truth” is unknown. Experimental studies are often unsuitable as benchmarks due to the convolution of several effects (e.g., surface restructuring, impurities at the electrode surface or in the electrolyte, mass transport effects, double layer effects, etc.). Therefore, using typical experimental results to validate simulations would require the consideration of all these effects, increasing the complexity of the model beyond what is currently possible. Perhaps an easier option would be to compare results with more accurate, albeit more expensive methods. However, in computational electrochemistry, such methods generally do not exist. For example, there is no atomic scale model that can accurately capture charge transfer processes within a realistic representation of the double layer. In fact, we do not even fully understand all the effects relevant to double layers.^93^

A second issue in defining a universal benchmark set is that different methods make substantially different approximations and often target different effects. Developing a balanced test set that all methods should pass therefore seems hard, if not impossible. Yet, it is exactly this wealth of different approximations that makes thorough benchmarking all the more important: otherwise the implications of certain approximations may not be recognized. The development of a universal test set may thus be ambitious, but there are good examples of tests/checks that could be performed for a wide class of methods used in computational electrochemistry. One example would be to check for the presence of pseudo-states that impact the computed reaction thermodynamics as described in the previous section.

At the workshop, it was broadly recognized that we as a community often fail to perform a thorough validation of methods used. We believe that this is a result of our strive to provide chemical insight into relevant processes and the societal urgency of these insights.

**Highlight 4:** To develop computational electrochemistry into a mature field and to avoid misinterpretations and incorrect conclusions, better method comparisons are needed—with benchmarks between different groups and to experiment whenever possible.

In particular, we believe that collaborative efforts in which different research groups approach the same question using different techniques (as sometimes published in the past) are extremely valuable. Such efforts, together with pedagogical studies that highlight the successes, failures, pitfalls, and opportunities of the different methods would serve as valuable guides for practitioners in the field.

Similar considerations also pertain to the computational set-ups used. In particular in atomistic modeling studies, we tend to make harsh practical decisions based on the computational cost. While this is often necessary, it is sometimes done without sufficient testing and without reporting the expected errors incurred due to these practical decisions.

For example, simulations aimed at estimating the potential of zero charge (PZC) of electrode–electrolyte interfaces are typically performed in small unit cells using the Γ-point only when sampling the Brillouin zone. For small (4 × 4) unit cells, this can lead to errors of several hundred meV in the work function (see Figure 5). These errors will directly translate into errors in the computed PZC but are often not discussed. Similarly, it is telling that it took until 2022 until the hot solvent–cold solute problem was discussed in the community.^95^ This thermostatting issue (that can occur for certain thermostats/thermostat settings) can cause the solvent to be hotter than the more rigid solute. Since the catalytic region can be considered as a ”solute”, this problem can impact interface simulations. Overall, we believe that some more rigor in the use of methods could be beneficial.

**Figure 5:**
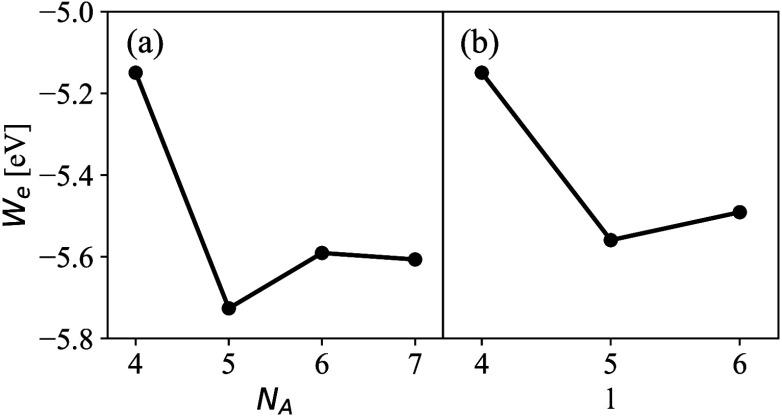
Dependence of the negative of the workfunction, *W*_e_, of Pt(100) on (a) the number of atoms, *N*_A_, in a *N*_A_ × *N*_A_ atom unit cell and (b) as a function of the number of layers *l* in the slab when only the Γ-point is considered during k-point averaging. The workfunction can change dramatically (several hundreds of meV) depending on the exact setup and can hence differ strongly from the true value. (*l* = 4 in panel a, and *N*_*A*_ = 4 in panel b.) This figure is reproduced from the Supporting Information of ref 94.

To close on a positive note, we would like to highlight some studies that *do* discuss such epistemic errors (i.e., errors caused by limited or inaccurate knowledge of the system). At the atomic scale, Calle-Vallejo et al.^96^ discussed errors in energies of gas-phase species incurred when using commonly used DFT exchange-correlation functionals and their influence on activity-volcano relationships of electrocatalysts. Kastlunger et al.^33^ and Hörmann et al.^34^ independently derived the equivalence of constant potential vs constant charge in the infinite cell size limit and identified various practical limitations in both approaches. Deißenbeck et al.^41^ compared the dynamics under constant potential conditions with that obtained when including charge-potential fluctuations. For microkinetic modeling, Reiher and co-workers investigated the uncertainties in complex reaction networks.^97^ Finally, the uncertainty in transport models has mainly been addressed in the fields of lithium-ion batteries^98,99^ and polymer electrolyte fuel cells.^100^ This list is far from exhaustive but highlights the valuable contribution of systematic studies on model uncertainties, as well as the impact of these uncertainties on the prediction of key properties. In the future, we anticipate that there will (and should) be more studies on epistemic errors in computational electrochemistry.

## Concluding Remarks

In summary, we have discussed the existing challenges and ongoing discussions in computational approaches to study electrochemical processes and, where appropriate, suggested a path forward.

The field of computational electrochemistry is relatively young and marked by a proliferation of methods. However, for computational electrochemistry to evolve toward a mature field, the strengths and limitations of the various methods need to be thoroughly understood, and clear guidelines for the various approaches need to be established. In doing so, we strongly believe that computational electrochemistry will play a crucial role in developing a thorough understanding of electrochemical processes from the atomistic to the system level.

## Footnotes

a E.g., a double-layer capacitance that is typically much lower than in experiments or in *ab initio* molecular dynamics simulations, the difficulty of smoothly connecting QM and MM water molecules, and the difficulty of charging up the QM cluster in response to changes in the MM part of the simulation cell.

b Note that some recent works show ESM-RISM^40^ to correctly predict the measured double layer capacitance.^43,44^

c Note that the magnitude of errors might be small, depending on the distance of the charged particle from the surface and the potential profile generated. We are unaware of rigorous tests published in this regard.

d After accounting for vibrational and rotational enthalpy and entropy effects.

e I.e., there is no need to subtract an additional term Δ*nμ*_*e*_(*U*) to account for the effective change in number of electrons Δ*n* required to keep the potential constant. In the CHE and constant charge methods, this is done by accounting for the electrons as reactants/products.

f We note that this scheme cannot be rigorously applied for barriers obtained from free energy sampling (e.g., slow-growth, metadynamics, etc.), as, in this case, the Gibbs free energy of the interface is unknown. Accessing it would require the use of computationally expensive insertion–deletion methods.^58^ One may, however, expect the influence of pseudo-states to be somewhat reduced as charges species can be brought further away from the surface. A rigorous proof for this statement is, to the authors knowledge, however missing to date.

g Note that the *y*-axis labels in several studies read *G*°. It should, however, be *G* for any species related to bulk concentrations and *G*° for adsorbed species, leading to a somewhat mixed representation.

h The symmetry factor is often approximated to be 0.5 but can also be estimated following a plethora of schemes.^56,57,66–68^

i E.g., if the coverage of reactants in the resting state is not constant or the resting state cannot be unambiguously identified.
